# A novel simplified scoring system for predicting mortality in emergency colorectal surgery: prediction model development

**DOI:** 10.1590/1516-3180.2018.0316240119

**Published:** 2019-07-15

**Authors:** Sadettin Er, Yusuf Sevim, Sabri Özden, Deniz Tikici, Barış Doğu Yıldız, Bülent Cavit Yüksel, Umut Fırat Turan, Mesut Tez

**Affiliations:** I MD. Attending Physician, Department of Surgery, Ankara Numune Eğitim ve Araştırma Hastanesi, Ankara, Turkey.; II MD. Associate Professor, Department of Surgery, Ankara Numune Eğitim ve Araştırma Hastanesi, Ankara, Turkey.; III MD. Attending Physician, Department of Surgery, Ankara Numune Eğitim ve Araştırma Hastanesi, Ankara, Turkey.; IV MD. Attending Physician, Department of Surgery, Ankara Numune Eğitim ve Araştırma Hastanesi, Ankara, Turkey.; V MD. Associate Professor, Department of Surgery, Ankara Numune Eğitim ve Araştırma Hastanesi, Ankara, Turkey.; VI MD. Associate Professor, Department of Surgery, Ankara Numune Eğitim ve Araştırma Hastanesi, Ankara, Turkey.; VII MD. Attending Physician, Department of Surgery, Ankara Numune Eğitim ve Araştırma Hastanesi, Ankara, Turkey.; VIII MD. Associate Professor, Department of Surgery, Ankara Numune Eğitim ve Araştırma Hastanesi, Ankara, Turkey.

**Keywords:** Colorectal surgery, Colorectal neoplasms, Mortality

## Abstract

**BACKGROUND:**

Despite advances in surgical approaches, emergency colorectal surgery has high mortality and morbidity.

**OBJECTIVE:**

We aimed to create a simple and distinctive scoring system, for predicting mortality among patients undergoing emergency colorectal surgery.

**DESIGN AND SETTING:**

Prediction model development study based on retrospective data-gathering.

**METHODS:**

Patients who underwent emergency colorectal surgery between March 2014 and December 2016 at a single tertiary-level referral center were included in our study. Patient demographics, comorbidities, type of surgery, etiology and laboratory and radiological findings were collected retrospectively and analyzed. A new clinical score (named the Numune emergency colorectal resection score) was constructed from the last logistic regression model, in which one point was assigned for the presence of each predictive factor.

**RESULTS:**

138 patients underwent emergency colorectal surgery. These comprised 64 males (46.4%) and 74 females (53.6%), with a mean age of 64 years. Multivariate analysis revealed that blood urea nitrogen level > 65 mg/dl (odds ratio, OR: 8.03; 95% confidence interval, CI: 2.16-15.77), albumin level < 0.7 ­mg/­dl (OR: 4.43; 95% CI: 1.96-14.39) and American Society of Anesthesiologists score ≥ 3 (OR: 3.47; 95% CI: 0.81-9.18) were associated with postoperative complications. The Numune score was graded from I to III. The risk of mortality was found to be 63.2% in the group with grade III, which accounted for 35.2% of the subjects. There were 37 postoperative deaths.

**CONCLUSIONS:**

Surgeons need scoring systems, especially to predict postoperative mortality. We propose the Numune emergency colorectal resection score for emergency surgical procedures as a practical, usable and effective system for predicting postoperative morbidity.

## INTRODUCTION

Despite advances in surgical approaches, emergency colorectal surgery has high mortality and morbidity.^1^ The mortality rates after emergency colorectal surgery range from 2.3% to 80%.^2^^,^^3^ This wide range is secondary to the expertise of the surgical center and the patients’ comorbidities. Colorectal emergency situations such as diverticulitis, trauma and ischemia may be related to either benign or malignant etiologies.^3^

Colorectal cancer is the reason behind colorectal emergencies in 85% of the cases, with colonic obstruction in 11%-43% of all presentations.^4^ Perforation and obstruction of the colon and rectum are important factors leading to postoperative mortality in patients with emergency admissions.^5^ The comorbidities that cannot be managed adequately in emergency colorectal surgery and which cause highest mortality are cardiopulmonary, renal and thromboembolic diseases.^6^

Scoring systems for use in predicting postoperative mortality after surgical procedures already exist. The Physiological and Operative Severity Score for the enUmeration of Mortality and morbidity (POSSUM) and the Portsmouth-Physiological and Operative Severity Score for the ­enUmeration of Mortality and morbidity (p-POSSUM) are two examples of such scoring systems. Both of these use physiological and operative parameters.^7^ Through use of these systems, it was realized that advanced age and high frequency of emergency procedures within colorectal surgery made these two scores inadequate. Thus, after omission of certain parameters, a new model was devised for colorectal surgery, which was named the colorectal-Physiological and Operative Severity Score for the enUmeration of Mortality and morbidity (cr-POSSUM).^8^

The cr-POSSUM system consists of multiple variables and defines a physiological score and an operative score. Followingthis, predictive values can be found through logarithmic equations. Computer software needs to be used in order to calculate predictive scores. The cr-POSSUM system can accurately predict postoperative complications, but unfortunately it is not practical, especially in emergency situations.

## OBJECTIVE

To propose a scoring system for predicting mortality among patients undergoing emergency colorectal surgery.

## METHODS

Local ethics board approval was obtained for this study, through registration number E-18-1938 (date: April 25, 2018). The patients included in this study underwent emergency colorectal surgery between March 1, 2014, and December 30, 2016, at a single tertiary-level referral center. The sociodemographic features, comorbidities, American Society of Anesthesiologists score, etiology, laboratory and radiological findings, blood transfusions, operative time, indications for surgery and type of surgery of the 138 patients thus included were analyzed retrospectively. Twenty-six patients whose data were incomplete were excluded from the study. Postoperative complications and mortality rates were also analyzed.

The following data were collected and used in the analyses as independent variables: age, gender, results from liver function tests (aspartate aminotransferase, alanine aminotransferase, alkaline phosphatase, gamma-glutamyl transferase and total and direct bilirubin), renal function tests, albumin level, complete blood cell count, type of surgery, presence of ostomy, operative time and blood transfusion. The primary endpoint (dependent variable) was postoperative mortality.

Continuous data were presented as the mean value ± standard deviation. Differences in continuous variables were analyzed using the Mann-WhitneyUtest. The Shapiro-Wilk test was used to assess normality of data distribution. Categorical variables were analyzed using chi-square tests. Logistic regression was used to identify the factors associated with mortality. The results from the multivariate analysis were presented as odds ratios with 95% confidence intervals. Receiver operating characteristic curve analyses were used to determine the optimal cutoff values for continuous variables.

A new clinical score (named the Numune emergency colorectal resection score) was constructed from the final logistic regression model, in which one point was assigned for the presence of each predictive factor. Model discrimination was measured as the area under the receiver operating characteristic curve. The discrimination of the prognostic model was considered perfect if the area under the curve was 1, good if the area under the curve was > 0.8, moderate if the area under the curve was 0.6-0.8 and poor if the area under the curve was < 0.6.^1^Specificity, sensitivity, positive predictive value, negative predictive value, negative likelihood ratio and positive likelihood ratio were also calculated.

## RESULTS

During the study period, emergency colorectal surgery was performed on 138 patients. These comprised 64 males (46.4%) and 74 females (53.6%), with a median age of 64 years (minimum: 23; maximum: 91).

The patients were classified according to their American Society of Anesthesiologists (ASA) score. Among them, 4 (2.90%) were classified as presenting ASA score I, 40 (28.99%) as ASA score II, 55 (39.86%) as ASA score III and 39 (28.26%) as ASA score IV. Twenty-three patients (16.7%) received blood transfusions during their surgery.

The most frequent surgical site was the right colon, followed in sequence by the sigmoid colon, the descending colon and the rectum. The average operative time was 147 ± 29 minutes (Table1). The most common indication for the surgery was obstruction, and the other indications were perforation and ischemia. The most common etiologies were colorectal cancer in 76 patients (55.07%), ischemic colitis in 27 patients (19.5%) and volvulus in 11 patients (7.97%) (Table 1).

**Table 1.** Demographic and clinical characteristics of patients

Variable	Survivors (n= 101)	Non-survivors (n= 37)	P
Mean age (years)	61 ± 14	72 ± 12	< 0.0001
Gender, male/female	53/48	12/25	0.0109
White blood cell count (/mm^3^)	13.07 ± 5.69	13.88 ± 7.44	0.0634
Aspartate aminotransferase(U/l)	20 (17-27)	139.63 (18-52)	0.005
Alanine aminotransferase(U/l)	14 (11-20)	148 (10-32)	0.718
Blood urea nitrogen (mg/dl)	39 (30-53)	70 (50-113)	< 0.0001
Creatinine (U/l)	0.97 (0.80-1.14)	1.33 (1.19-1.99)	< 0.0001
Albumin (mg/dl)	3 (2.6-3.4)	2.55 (2.1-2.8)	0.003
American Society of Anesthesiologists score ≥ 3	60	25	< 0.0001
Etiology			
Colorectal cancer	66	10	< 0.0001
Ischemia	8	19	
Volvulus	8	3	
Diverticulitis	6	1	
Other	13	4	
Type of surgery			
Right hemicolectomy	47	17	0.613
Left hemicolectomy	16	5	
Low anterior resection	10	5	
Sigmoid resection	10	4	
Transvers colectomy	2	0	
Anterior resection	11	2	
Total colectomy	2	3	
Subtotal colectomy	3	1	
Operation time	149 ± 27	140 ± 29	0.5
Preventive ostomy +/-	57/44	29/8	0.028
Reason for mortality			
Sepsis	0	17	
Multiple organ failure	0	12	
Pneumonia	0	6	
Pulmonary thromboembolism	0	2	

All the operations were performed by general surgeons. Regarding the type of surgery, right hemicolectomy was conducted in the cases of 64 patients (46.9%), followed by left hemicolectomy in 21 (15.2%), low anterior resection in 15 (10.9%), anterior resection in 13 (9.4%), total abdominal colectomy in 5 (3.6%) and subtotal abdominal colectomy in 4 (2.9%). A preventive ostomy was created in 86 patients (62.3%).

Thirty-seven postoperative deaths occurred (26.8% of the patients). The main reason for mortality was sepsis, which occurred in the cases of 17 patients (11.6%). The other reasons for mortality comprised multiple organ failure in 12 patients (8.2%), pneumonia in 6 (4.1%) and pulmonary thromboembolism in 2 (1.4%) (Table1). There were 38 occurrences of minor complications among the patients. Surgical wound infection was the most common minor complication (15.9%). Other reasons for occurrences of wound infection included bleeding in 3 patients (2.1%), anastomotic leakage in 7 (4.8%), necrosis of ostomy in 3 (2.1%) and postoperative ileus in 1 (0.7%).

In univariate analyses, age greater than 65 years, American Society of Anesthesiologists score greater than or equal to 3, blood urea nitrogen level higher than 65 mg/dl, creatinine higher than1.2­mg/­dl, albumin level lower than 2.7 mg/dl, aspartate aminotransferase level higher than 50 u/ml and indication for resection were found to be statistically associated with postoperative mortality (Table 1).

### Multivariate risk prediction model and prediction score

All of the variables that could be assessed before the operation were included in the multivariate model. Three variables were found to be significant in this analysis: blood urea nitrogen level >65 mg/dl (odds ratio, OR: 8.03; 95% confidence interval, CI:2.16-15.77); albumin level < 0.7 mg/dl (OR: 4.43; 95% CI:1.96-14.39); and American Society of Anesthesiologists score ≥ 3 (OR: 3.47; 95% CI: 0.81-9.18) (Table 2).

**Table 2.** Multivariate logistic regression model for predictors of mortality

	Regression coefficient	Odds ratio	95% confidence interval	P	Score points
American Society of Anesthesiologists score ≥ 3	1.247	3.478	0.81-9.18	0.048	1
Blood urea nitrogen level> 50 mg/dl	2.083	8.032	2.16-15.77	0.001	1
Albumin level < 2.7 mg/dl	1.7	5.48	1.96-14.39	0.0006	1

A probability score was calculated by adding together the number of points assigned to each variable. Although the regression coefficients ranged from 1.24 to 2.08, one point was assigned to each of these risk factors, for simplicity. The resulting Numune emergency colorectal resection score (composed of the blood urea nitrogen level, albumin level and American Society of Anesthesiologists score) was graded on a range from I to III.

Three groups of patients were defined based on the Numune emergency colorectal resection score. The first group, with a score graded as I, comprised 64.8% of the patients, and their mortality rate was 7.1%. The second group included patients with a score graded as II, who had a mortality rate of 26.6%; this group comprised approximately 28% of the cohort. The third group, which comprised approximately 35.2% of the patients, included those with a Numune emergency colorectal resection score graded as III, and their mortality rate was 63.15% (Table 3).

**Table 3.** Risk of mortality according to the Numune emergency colorectal resection score

Numune emergency colorectal resection score		Mortality rate(%)
Grade	Score points	
I (70 patients)	0	5 (7.14)
II (30 patients)	1	8 (26.6)
III (38 patients)	≥ 2	24 (63.15)

**Figure 1.**
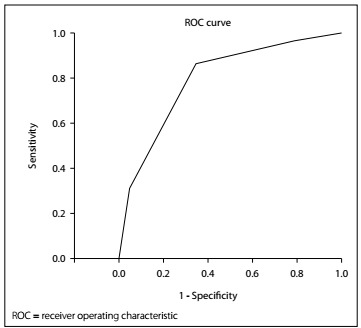
The area under the receiver operating characteristic curve (AUROC) was 0.794 (95% confidence interval, CI: 0.704-0.885) for the Numune emergency colorectal resection score.

The specificity, sensitivity, positive predictive value, negative predictive value, negative likelihood ratio and positive likelihood ratio for the Numune emergency colorectal resection grades of II or III were 64.36%, 86.49%, 47.06%, 92.86%, 0.21 and 2.43, respectively. The area under the receiver operating characteristic curve was 0.794 (95% CI: 0.704-0.885) for the Numune emergency colorectal resection score (Figure 1).

## DISCUSSION

Prediction of postoperative mortality after emergency colorectal surgery is an ongoing field of research. In this study, we defined a new scoring system, the Numune emergency colorectal resection score. The simplicity of our calculation is quite apparent, as it uses only three variables, among which two are part of the routine serum biochemistry evaluation: blood urea nitrogen level, albumin level and American Society of Anesthesiologists score.

Several scoring systems are used within colorectal surgery to predict postoperative morbidity and mortality. The Physiological and Operative Severity Score for the enUmeration of Mortality and morbidity (POSSUM) was designed by Copeland etal. in order to evaluate postoperative morbidity and mortality.^9^ The Portsmouth-Physiological and Operative Severity Score for the enUmeration of Mortality and morbidity (P-POSSUM) and the colorectal-Physiological and Operative Severity Score for the enUmeration of Mortality and morbidity (cr-POSSUM) were designed based on the POSSUM scoring system.

The most recent of these scoring systems, i.e. cr-POSSUM, consists of 10 parameters (six for the physiological severity score and four for the operative severity score), and the final score is calculated by means of a logarithmic equation.^10^ Although some medical electronic software for calculating this score exists, this scoring system is impractical, given that it necessitates greater effort and more data. Moreover, it has been reported that cr-POSSUM may either overestimate or underestimate mortality.^11^^,^^12^

Heriot etal. studied prediction of postoperative morbidity and mortality among elderly patients who underwent colorectal surgery, and they designed the elderly colorectal cancer model.^1^ However,this scoring system has not undergone any external validation. In addition, the Association of Coloproctology of Great Britain and Ireland Malignant Large Bowel Obstruction model was generated to evaluate in-hospital mortality. This consists of four parameters.^14^ It may be practical to use but, like the elderly colorectal cancer model, it has not been externally validated.

Furthermore, Sluis etal. published the Identification of Risk in Colorectal Surgery score. They stated that their scoring system presented greater discriminatory capacity than that of the cr-POSSUM scoring system and that of the American Society of Anesthesiologists score classification for predicting postoperative mortality.^15^ The Identification of Risk in Colorectal Surgery scoring system may be useful generally in colorectal surgery.

Our scoring system consists of three parameters, and the physiological status of the patients is evaluated by means of the American Society of Anesthesiologists score classification. The Numune emergency colorectal resection score is useful in emergency procedures, and is simple to use.

The scoring system of the French Association of Surgery (Association Française de Chirurgie, AFC) uses four simple parameters to predict postoperative mortality, comprising intraoperative fecal contamination, operative time greater than six hours, American Society of Anesthesiologists score > 2 and smoking.^16^ These parameters were found to be independent risk factors for mortality after colorectal surgery. However, the Elderly-Physiological and Operative Severity Score for the enumeration of Mortality and Morbidity was found to be better than the AFC scoring system for predicting postoperative mortality after colorectal surgery.^17^ Weused the American Society of Anesthesiologists score, blood urea nitrogen level and albumin level in the Numune emergency colorectal resection score, and these are helpful for preoperatively predicting the postoperative mortality.

Another scoring system that has been used to predict postoperative morbidity and mortality is the American College of Surgeons surgical risk calculator.^18^ The colon-specific model of this scoring system uses multiple factors affecting postoperative morbidity and mortality. This surgical risk calculator may help surgeons to estimate patient-specific postoperative risks but, again, it requires too many parameters, in comparison with the Numune emergency colorectal resection score.

Some medical calculators or scoring systems are available electronically. These software programs may help in using the scoring systems correctly, but are more useful in elective surgical procedures. For emergency surgery, systems that are more practical and easier to use, like the Numune emergency colorectal resection score are more useful.

However, one shortcoming of our scoring system is that it contains the American Society of Anesthesiologists score, which is subjective and can give rise to inter-observer variability.^19^ Ourscoring system may help clinicians to select high-risk patients for transfer to an advanced center.

There are some limitations to our study. We did not have any control group, and so the positive predictive value of the study was low. The analyses were not assessed regarding the underlying indications or type of surgery. All of these are shortcomings of the study. The patients’ ages were found to be significant only in the univariate analyses. This may have been related to the distribution of the ages of the patients included in our study. Futurestudies may reveal that age is an important parameter and, if so, our scoring system may need to be updated.

## CONCLUSION

Surgeons need scoring systems or some parameters in order to predict postoperative morbidity and, especially, mortality. Therefore, researchers need to make efforts towards devising optimal scoring systems for use both in elective and in emergency surgery. The Numune emergency colorectal resection score for use in relation to emergency surgical procedures seems to be an option for predicting postoperative mortality among patients undergoing emergency colorectal surgery.
